# Involvement of Opioid System and TRPM8/TRPA1 Channels in the Antinociceptive Effect of *Spirulina platensis*

**DOI:** 10.3390/biom11040592

**Published:** 2021-04-17

**Authors:** Mariana A. Freitas, Amanda Vasconcelos, Elaine C. D. Gonçalves, Eduarda G. Ferrarini, Gabriela B. Vieira, Donatella Cicia, Maíra Cola, Raffaele Capasso, Rafael C. Dutra

**Affiliations:** 1 Laboratory of Autoimmunity and Immunopharmacology (LAIF), Department of Health Sciences, Campus Araranguá, Universidade Federal de Santa Catarina, Araranguá 88906-072, Brazil; freitas.mariana@grad.ufsc.br (M.A.F.); amanda.vasconcelos@grad.ufsc.br (A.V.); cristina.cdg@posgrad.ufsc.br (E.C.D.G.); eduarda.ferrarini@posgrad.ufsc.br (E.G.F.); gabriela.b.v@posgrad.ufsc.br (G.B.V.); maira.cola@ufsc.br (M.C.); 2 Post-Graduate Program of Neuroscience, Center of Biological Science, Campus Florianópolis, Universidade Federal de Santa Catarina, Florianópolis 88040-900, Brazil; 3 Department of Pharmacy, University of Naples Federico II, 80131 Naples, Italy; donatella.cicia@unina.it; 4 Department of Agricultural Sciences, University of Naples Federico II, 80055 Portici, Italy; 5 Endocannabinoid Research Group, 80078 Pozzuoli, Italy

**Keywords:** *Spirulina platensis*, pain, opioid system, ionic channel, functional food, analgesic

## Abstract

*Spirulina platensis* is a “super-food” and has attracted researchers’ attention due to its anti-inflammatory, antioxidant, and analgesic properties. Herein, we investigated the antinociceptive effects of *Spirulina* in different rodent behavior models of inflammatory pain. Male Swiss mice were treated with *Spirulina* (3–300 mg/kg, p.o.), indomethacin (10 mg/kg, p.o.), or vehicle (0.9% NaCl 10 mL/kg). Behavioral tests were performed with administration of acetic acid (0.6%, i.p.), formalin 2.7% (formaldehyde 1%, i.pl.), menthol (1.2 µmol/paw, i.pl.), cinnamaldehyde (10 nmol/paw, i.pl.), capsaicin (1.6 µg/paw, i.pl.), glutamate (20 µmol/paw, i.pl.), or naloxone (1 mg/kg, i.p.). The animals were also exposed to the rotarod and open field test to determine possible effects of *Spirulina* on locomotion and motor coordination. The quantitative phytochemical assays exhibited that *Spirulina* contains significant concentrations of total phenols and flavonoid contents, as well as it showed a powerful antioxidant effect with the highest scavenging activity. Oral administration of *Spirulina* completely inhibited the abdominal contortions induced by acetic acid (ED_50_ = 20.51 mg/kg). *Spirulina* treatment showed significant inhibition of formalin-induced nociceptive behavior during the inflammatory phase, and the opioid-selective antagonist markedly blocked this effect. Furthermore, our data indicate that the mechanisms underlying *Spirulina* analgesia appear to be related to its ability to modulate TRMP8 and TRPA1, but not by TRPV1 or glutamatergic system. *Spirulina* represents an orally active and safe natural analgesic that exhibits great therapeutic potential for managing inflammatory pain disorders.

## 1. Introduction

Functional foods are hopeful for good health and an essential part of a healthy lifestyle [1]. According to Henry [2], the idea around functional foods follows the oriental philosophy that established “medicine and food have a common origin” [2]. In this context, when associated with a balanced diet and physical activity, functional foods’ consumption could be linked to beneficial health effects [3,4]. It includes their ability to reduce heart disease risk, mitigate neurodegenerative diseases, and prevent metabolic disorders such as type 2 diabetes mellitus (T2DM) [5,6,7]. Despite its benefits, functional foods still lack definition since this term has not been universally accepted [3]. However, it is possible to characterize them with active components, consumed as part of the usual diet, and they have been scientifically proven to provide a health benefit beyond essential nutrition [3,8]. Indeed, diets based on vegetable origin foods are considered excellent sources of ingredients [8,9], including omega-3 (ω-3), omega-6 (ω-6), omega-9 (ω-9), tocopherols, phenolic acids, beta-carotene, and others, which mediate anti-inflammatory and antioxidant effects through the activation of multiple cellular pathways [10,11]. In this way, previous studies demonstrated that functional food consumption can activate, for example, peroxisome proliferator-activated receptors (PPAR)—a nuclear transcription factor involved in lipid metabolism—which has been shown to prevent dyslipidemia and obesity [6,12,13,14]. Functional foods and their bioactive substances also affect gut homeostasis and stimulate the growth of specific intestinal microbiota members [15,16]. Short-chain fatty acids (SCFAs) derived from dietary fiber’s fermentation can bind to the ‘metabolite sensing’ receptor, such as G-protein-coupled receptor 43 (GPR43) on colonic epithelial cells, leading to NLR family pyrin domain-containing 3 (NLRP3) inflammasome activation [15]. In turn, the inflammasome pathway mediates the release of both the pro-inflammatory cytokines IL-1β and IL-18, providing protection against bacterial, viral, and protozoal infections [17]. Plant foods naturally rich in polyphenols such as strawberry and cranberry also improved insulin sensitivity in overweight and obese non-diabetic, insulin-resistant human subjects [18]. Still, according to Alkhatib [19], functional foods could optimize the immune system capacity to prevent and control pathogenic viral infections [19]. Altogether, it is possible to understand that functional foods can be considered essential tools in maintaining a healthy lifestyle and contributing to the prevention and treatment of different disorders. However, these effects’ underlying mechanisms have not yet been fully elucidated and need to be studied.

In this context, great attention has been focused on *Spirulina platensis*, a filamentous cyanobacterium used as a supplement food with highly nutritious potential feed resources and humanitarian instruments in fighting against severe malnutrition worldwide [10,20], because it is not expensive and is considered by the World Health Organization (WHO) as one of the most curative and prophylactic components of nutrition in the twenty-first century, and for this reason, some researchers have even recognized it as “super-food” [20,21]. In turn, some animal and human studies have reported anti-inflammatory, antioxidant, and immunomodulatory properties linked to this microalga [10]. These effects are associated with its rich nutrient *Spirulina* composition, including proteins (65%), minerals (7%), carbohydrates (20%), fatty acids (5–7%), essential amino acids, vitamins (vitamin B1, vitamin B12), antioxidants, γ-linolenic acid, chlorophyll, carotenoids, and phycocyanin [10,22,23,24], although its exact mechanism of action still remains unclear. In such a way, given its high nutritional value, the medicinal properties of *Spirulina* have been previously investigated. A recent study demonstrated that streptozotocin (STZ)-induced diabetic rats given oral *Spirulina* (500 mg/kg/day, for one month) showed lower pain scores in a formalin test when compared to the control group (untreated), suggesting that *Spirulina* could modulate nociceptive pathways [25]. Reddy and colleagues reported that C-phycocyanin-pigment-binding protein isolated from *Spirulina* is a selective inhibitor of cyclooxygenase-2 (COX-2), a mechanism by which the microalga could exert its anti-inflammatory and antinociceptive activity [26]. Moreover, immunohistochemical data from 6-hydroxydopamine (6-OHDA)-lesioned rat’s striatum showed that treatment with *Spirulina* (50 mg/kg, p.o.) significantly reduced inducible nitric oxide synthase (iNOS) and COX-2 immunoreactivity [27]. Another study also showed that *Spirulina* exerts analgesic effects in rats exposed to acetic acid-induced writhing response and hot plate test, associated with reduced IL-6, tumor necrosis factor (TNF), IL-1β, nitric oxide (NO) levels, and prostaglandin E(2) (PGE2), and suppressed the activities of COX-2 and iNOS [28]. However, there is still a lack of literature about the *Spirulina* mechanisms underlying anti-inflammatory and antinociceptive activities [28]. One way to evaluate the involvement of possible mechanisms involved in nociceptive responses is to use a test such as formalin that acts specifically on primary afferent sensory neurons through a direct action on transient receptor potential ankyrin 1 (TRPA1), a member of the potential transient receptor family of cationic channels that is highly expressed by a subset of C fiber nociceptors [29]. Keeping the above data in mind, the purpose of the present study was to investigate the *Spirulina* antinociceptive effect and its mechanism of action related to ascending pain control.

## 2. Materials and Methods

### 2.1. Drugs and Reagents

The following materials were used: acetic acid and absolute ethyl alcohol (Neon, São Paulo, Brazil), L-glutamic acid (glutamate) (Vetec, Rio de Janeiro, Brazil), capsaicin, cinnamaldehyde, indomethacin (Sigma-Aldrich, St. Louis, MO, USA), ketamine hydrochloride, xylazine hydrochloride (Syntec, São Paulo, Brazil), naloxone hydrochloride (Cristália, São Paulo, Brazil), formalin (Dinâmica, São Paulo, Brazil), sodium hydroxide (Nuclear, São Paulo, Brazil), menthol (A Essência, Santa Catarina, Brazil), and tween 80 (Labsynth, São Paulo, Brazil). The drugs were dissolved in 0.9% NaCl solution (saline) before administration, except cinnamaldehyde (1% tween 80 in saline), glutamate (saline solution at pH 7), and menthol (1.6% absolute ethyl alcohol + 0.01% tween 80 in saline). Capsaicin was prepared from a 0.5% capsaicin stock solution dissolved in absolute ethyl alcohol, and the 0.1% solution was prepared at the time of use by mixing the stock solution with tween 80 and 2:1:7 saline solution.

### 2.2. Plant Material and Extract Preparation

Powdered biological *Spirulina*, originally from China—Certiplanet PTBIO-04 [29], was obtained from Iswari^®^, Portugal, dissolved in 0.9% NaCl solution at room temperature. The composition of the *Spirulina* was provided by the supplier and is shown in Table 1.

In a new set of experiments, 2 g of *Spirulina* dried powder were weighted in a glass vial and extracted with 20 mL of water by a heating plate at 45 °C for 60 min under shaker. The mixture was centrifuged at 3000 rpm for 10 min (Fanem 002CB, São Paulo, Brazil), and then filtered through a filter paper. The extraction process was carried out in triplicate. The obtained filtered extracts were used for determination of total phenolic compounds, total flavonoid compounds, and antioxidant activity [30,31,32].

### 2.3. Total Phenolic Compound Determination

The concentration of total phenolic compounds in *Spirulina* was determined according to the Folin–Ciocalteu method [33,34]. Briefly, samples (200 μL) were introduced into test tubes in which 1 mL of Folin–Ciocalteu’s reagent (previously diluted 10× with water) and 1 mL of sodium carbonate (20%, *w/v*) were added. The tubes were mixed and allowed to stand in darkness at room temperature for 30 min. Absorption at 765 nm (for gallic acid) and 720 nm (for chlorogenic acid) against a blank was measured using anultraviolet–visible (UV/VIS) Spectrophotometer (Global Trade Technology UV GT7220, São Paulo, Brazil). A blank sample of extract was used for background subtraction. The total phenolic content was expressed as mg/mL gallic acid or chlorogenic acid equivalents using a calibration curve. All measurements were carried out in triplicate and expressed as mean ± standard deviation (SD).

### 2.4. Flavonoid Compound Determination

Flavonoid content was assessed according to the procedure of Dutra et al. [35] based on the aluminum chloride complex formation. To 1 mL of *Spirulina* extract, 0.1 mL of 10% (*w/v*) AlCl3 methanolic solution and 0.1 mL of 5% (*w/v*) NaNO_2_ solution were added. The mixture was allowed to react for 11 min at room temperature and the absorbance was read at 420 nm (for quercetin) and 510 nm (for catechin) against a blank. A blank sample of extract was used for background subtraction. The flavonoid content was expressed as mg/mL quercetin or catechin acid equivalents using a calibration curve. All measurements were carried out in triplicate and expressed as mean ± standard deviation (SD).

### 2.5. Antioxidant Activity

The improved Trolox Equivalent Antioxidant Capacity (TEAC) assay was used [36]. Briefly, a solution of 2,2-diphenyl-1-picrylhydrazyl (DPPH) (125 mM) in ethanol and 0.05 mol/L acetate buffer (pH 5.5) were prepared. A dilution 1/5 of the extracts was performed for the assay in order to fall in the linear range of the response. The mixture was vortexed immediately after adding DPPH and allowed to stand at room temperature in a dark environment for 30 min, and the absorbance was read at 517 nm by a spectrophotometer. The antioxidant activity was obtained using a standard curve plotted with varying concentrations of Trolox (0 to 250 μg/mL). Determinations were made in triplicate and results were expressed as %Trolox equivalent (μg/mL)/100 g of *Spirulina*. All data are expressed as mean ± standard deviation (SD).

### 2.6. Animals

The experiments were performed on a total of 218 male Swiss mice (40–50 g, 60–90 days of age) obtained from the Universidade Federal de Santa Catarina (UFSC). Animals (maximum of 12 mice per group, housed in clear, transparent plastic cages with dust-free sawdust bedding) were kept under a 12 h light/dark cycle (artificial light on at 7:00 a.m.) and temperature (22 ± 2 °C). They were fed a pelleted and extruded mouse diet ad libitum and had unrestricted access to drinking water. Animals were acclimatized to laboratory settings for at least 1 h before testing and were used only once throughout the experiments. Mice were randomly assigned before treatment or behavioral evaluation. The treatment and evaluations were always performed at the same time and respecting the light/dark cycle. All procedures in this study were performed following the National Institute of Health Guide for the Care and Use of Laboratory Animals [37] and were approved by the Animal Ethics Committee of the Universidade Federal de Santa Catarina (CEUA-UFSC, protocol number 3914220319—approved on 10 May 2018). The mice were randomly assigned to groups before treatments, and behavioral evaluations were performed between 8:00 a.m. and 5:00 p.m. All behavioral analyses were measured manually, and the observer was blinded to the experimental protocols. Moreover, the number of animals and the intensity of the noxious stimuli used were the minima necessary to demonstrate consistent effects. All of the experimental procedures were conducted according to the guidelines of CONCEA and CEUA/UFSC, based on the principles of the 3Rs (Replacement, Reduction, and Refinement).

### 2.7. Antinociceptive Activity

#### 2.7.1. Abdominal Writhing by Intraperitoneal Injection of Acetic Acid

**Figure 1:**
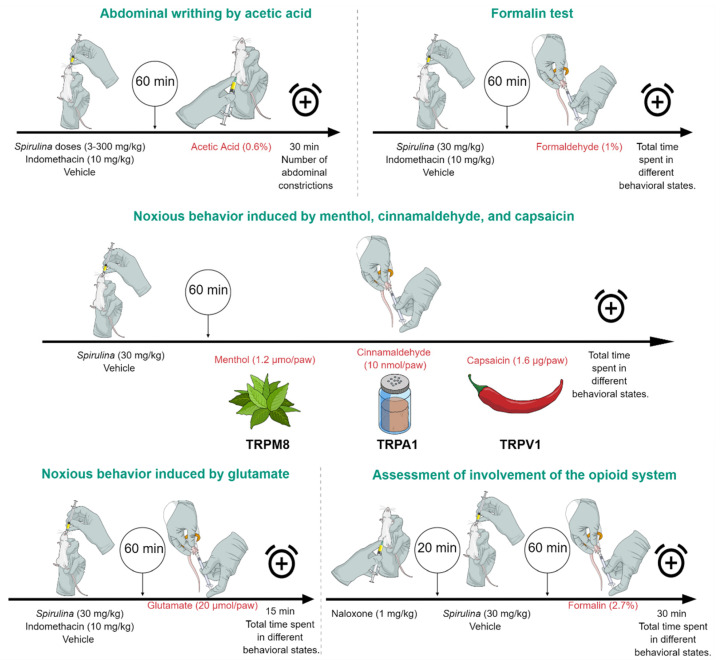
Experimental design. The acetic acid abdominal writhing protocol evaluated the pretreatment with *Spirulina* (3–300 mg/kg, p.o.), indomethacin (10 mg/kg), or vehicle (0.9% NaCl 10 mL/kg), and after 60 min, 0.6% acetic acid was administered via i.p. to assess the nociceptive behavior. In the formalin test, its application occurred viai.pl. after 60 min of treatment and the time spent in different behavioral states was evaluated. The harmful behavior induced by the menthol, cinnamaldehyde, and capsaicin protocol was administered i.pl. after 60 min of treatment and aimed to assess the involvement of TRPM8, TRPA1, and TRPV1, respectively. The glutamatergic system’s involvement was analyzed with the administration of glutamate (20 µmol/paw) via i.pl. and the time spent in different behavioral states was evaluated for 15 min. To assess the involvement of the opioid system, a pretreatment with naloxone (1 mg/kg, i.p.) was administrated 20 min before treatment with *Spirulina* (30 mg/kg, p.o.), and after 60 min, nociceptive behaviors were evaluated.

The abdominal constriction induced by intraperitoneal injection of acetic acid (0.6%) was carried out according to the procedures described previously by Beirith et al. [38]. Animals were pre-treated with different *Spirulina* doses (3–300 mg/kg, p.o.) or indomethacin (10 mg/kg, p.o., used as positive control) 60 min before testing. The vehicle group received 0.9% of NaCl (10 mL/kg, p.o.). After the challenge, mice were placed in separate boxes, and the number of abdominal constrictions was cumulatively counted for 30 min [39] (Figure 1).

#### 2.7.2. Formalin Test

The procedure used was similar to that described previously from Hunskaar and Hole [40]. In this model, two distinct periods can be evaluated, the neurogenic period, called the early phase (Phase I), identified in the first 5 min of the test, and the inflammatory period, named as late phase (Phase II), lasting 20 to 30 min after the injection of formalin [39]. In this way, 20 µL of 2.7% formalin solution (1% formaldehyde) was injected under the skin of the dorsal surface of the right hind paw. The animals were pre-treated with *Spirulina* (30 mg/kg, p.o.) or indomethacin (10 mg/kg, p.o., used as a positive control) 60 min before the test. The vehicle group received 0.9% NaCl (10 mL/kg). After the injection of the formalin solution, the animals were evaluated for 30 min (Figure 1). The quantification was based on the total time spent in different behavioral states, timed with a chronometer, and considered indicative of hyperalgesia, which was characterized by the animal elevating, licking, biting, or shaking the injected paw, or reducing the weight put on it.

### 2.8. Open Field and Rotarod Test

To assess whether *Spirulina* could change the locomotor activity of animals, an open field test was carried out, in which the numbers of crossings (indicative of locomotor activity) were evaluated [41]. Thus, the animals were pre-treated with *Spirulina* (30 mg/kg) or indomethacin (10 mg/kg, p.o.) 1 h before the test. Control animals received 0.9% NaCl (10 mL/kg). In this test, mice were placed individually in a wooden box (40 × 60 × 50 cm^3^) with the floor divided into 12 squares to investigate locomotion activity during 5 min, and the crossing number (number of squares crossed by the animal using all paws) was used to evaluate locomotion activity. Additionally, the rotarod test was applied to assess interferences in coordination and motor planning [42]. The protocol was developed at a speed of 32 rpm, over 60 s, making it possible to verify the latency period related to the number of falls [43]. The animals were pre-treated with *Spirulina* (30 mg/kg) or vehicle (0.9% NaCl 10 mL/kg) 1 h before the evaluation.

### 2.9. Noxious Behavior Induced by Menthol, Cinnamaldehyde, and Capsaicin

To verify the involvement of the Transient Receptor Potential channels (TRP channels)—subfamily M (melastatin) member 8 (TRPM8), subfamily A (ankyrin) member 1 (TRPA1), and subfamily V (vanilloid) member 1 (TRPV1)—in *Spirulina*’s antinociceptive activity, the effects of this microalga against nociceptive responses triggered by specific activators of these channels were tested, as described previously [44]. In these tests, the animals were pre-treated with *Spirulina* (30 mg/kg, p.o.) or vehicle (0.9% NaCl 10 mL/kg, control group), 1 h before the algogenic injections. In this test, menthol (1.2 µmol/paw, i.pl.), cinnamaldehyde (10 nmol/paw, i.pl.), and capsaicin (1.6 µg/paw, i.pl.) were used to evaluate the involvement of the TRPM8, TRPA1, and TRPV1 channels, respectively (Figure 1). Quantification of noxious behavior was based on the total time spent in different behavioral states characterized by the animal elevating, licking, biting, or shaking the injected paw or reducing the weight put on it in each test 20 or 5 min after menthol and cinnamaldehyde/capsaicin administration, respectively.

### 2.10. Noxious Behavior Induced by Glutamate

The protocol evaluated the possible interaction of *Spirulina* with the glutamatergic system described previously by Meotti et al. [45]. Briefly, mice were pre-treated with *Spirulina* (30 mg/kg, p.o.) or indomethacin (10 mg/kg, p.o.) 1 h before the glutamate injection, and control mice received 0.9% NaCl (10 mL/kg). The total time spent elevating, licking, biting, or shaking the injected paw or reducing the weight put on it was recorded in the initial 15 min of evaluation after the intra-plantar injection of glutamate (20 µmol/i.pl.) (Figure 1). The sum of seconds accumulated in different behavioral states was chosen to represent nociception induced by all solutions.

### 2.11. Assessment of Involvement of the Opioid System

To investigate the involvement of the opioid system in the analgesic effect of *Spirulina*, in this set of experiments, mice were pre-treated with naloxone (1 mg/kg, i.p.) [38]. After 20 min, the animals received *Spirulina* (30 mg/kg, p.o.) or vehicle (0.9% NaCl 10 mL/kg). After 1 h, animals received injection of formalin (2.7%). After the formalin solution injection, the animals were evaluated for 30 min (Figure 1). The quantification was based on the total time spent in different behavioral states, timed with a chronometer and considered indicative of hyperalgesia, which was characterized by the animal elevating, licking, biting, or shaking the injected paw or reducing the weight put on it (Figure 1).

### 2.12. Statistical Analysis

Non-clinical data are expressed as mean ± standard error of the mean (SEM) of 3–6 mice per group and represent two independent experiments (n_total_ = 6–12 animals/group). Normality and homoscedasticity were evaluated using Shapiro–Wilk’s and Levene’s tests, respectively. Repeated measurements were considered within-subject random factors, and all results were analyzed by a mixed-model one-way analysis of variance (ANOVA). The effective dose (ED_50_) value (i.e., the dose of *Spirulina* producing half-maximal antinociceptive or the dose necessary to reduce the nociceptive response by 50% relative to the control value) was calculated by nonlinear regression analysis and reported as the geometric mean. The percentages of inhibition were obtained in each experiment concerning the control values (vehicle-treated mice for nociception tests, 100% response obtained with the vehicle). All experiments were performed by a blind operator concerning nociception assessments and statistical analyses. A statistical comparison of the data was performed by one-way ANOVA followed by Newman–Keuls’ test. *p*-values of <0.05, <0.01, and <0.001 were considered statistically significant. Statistical analyses were performed using GraphPad Prism 8.2.1 software (GraphPad Software Inc., San Diego, CA, USA).

## 3. Results

### 3.1. Total Phenol and Flavonoids Contents, and Antioxidant Effect

The content of total phenols and flavonoids in *Spirulina* were determined spectrometrically using the Folin–Ciocalteu reagent and aluminum chloride complex formation, respectively (Table 2). Moreover, Table 2 contains the results of antioxidant activity determined using DPPH, expressed as %Trolox equivalent (μg/mL)/100 g of *Spirulina*. The correlation between the phenolic and flavonoid contents of *Spirulina* and its corresponding antioxidant activity were also determined (0.90 and 0.82), respectively. Taken together, our data suggest that the radical scavenging effect is related to both the quantity and efficiency of the type of phenolic compound (i.e., gallic acid and chlorogenic acid) and flavonoids (quercetin and catechin) present in the samples.

### 3.2. Nociceptive Behavior Induced by Acetic Acid

**Figure 2:**
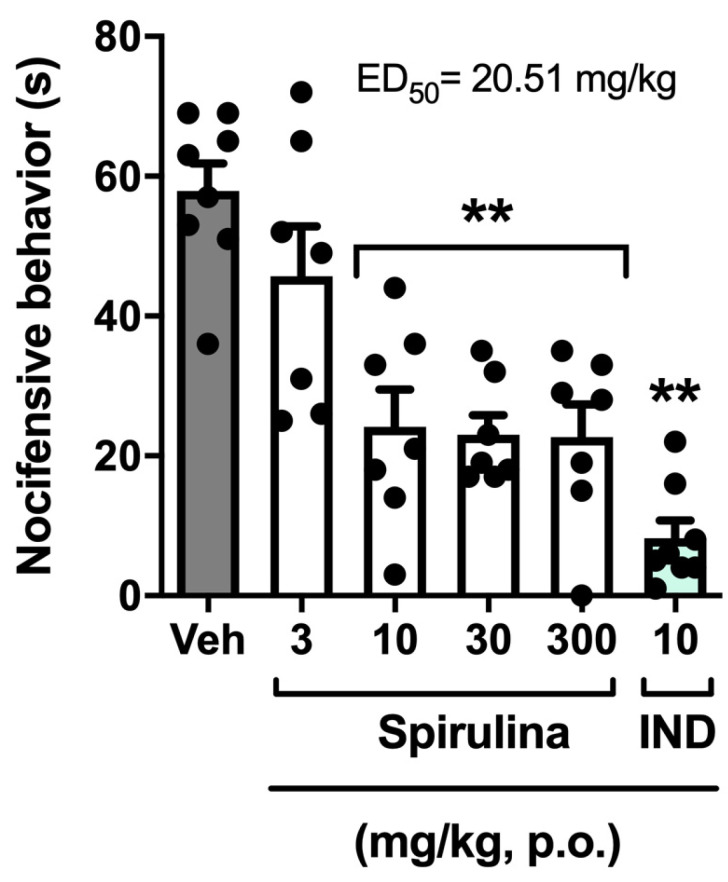
Effect of *Spirulina* on nocifensive behavior induced by intraperitoneal injection of acetic acid in mice. Each column represents the mean ± SEM of 7–8 animals/group, with a black dot showing individual mice. Asterisks denote the levels of significance compared to the vehicle group (Veh). ** *p* < 0.001 using one-way ANOVA. IND: indomethacin.

Oral treatment with *Spirulina* (3, 10, 30, and 300 mg/kg) significantly inhibited the number of abdominal writhing induced by acetic acid when compared to the control group (Figure 2), with the percentage of inhibition of 49.76%, 72.76%, 52.58%, and 62.91%, respectively. Indomethacin (10 mg/kg, p.o.), used as a positive control, inhibited the abdominal writhing response, with a rate of 90.14% (Figure 2). Regression analysis showed that *Spirulina* has an ED_50_ of 20.51 mg/kg and reached the maximal effect (E_max_) at a dose of 254.60 mg/kg, as evidenced in the dose–effect graphic (Figure 2). Therefore, it was possible to observe that, regardless of the dose, *Spirulina* showed an analgesic effect in the abdominal writhing test similar to the positive control. After this set of experiments, the dose of 30 mg/kg of *Spirulina* was used in subsequent experiments to investigate some of the mechanisms that underlie its anti-hyperalgesic properties.

### 3.3. Nociceptive Behavior Induced by Formalin

**Figure 3:**
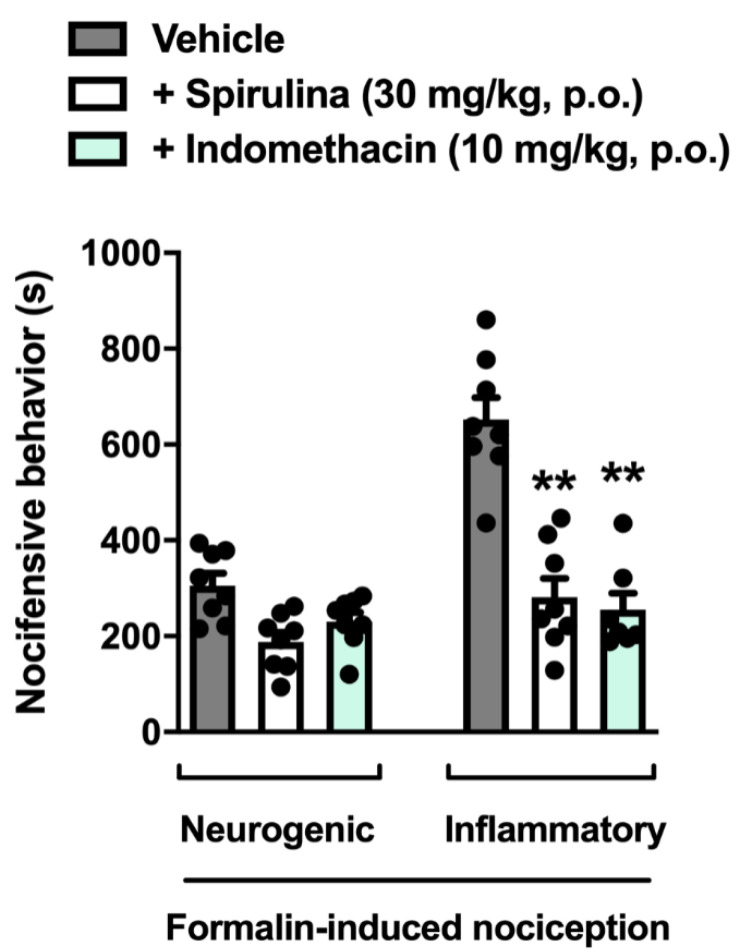
Effect of *Spirulina* on nocifensive behavior induced by formalin in mice during the first (neurogenic) and second (inflammatory) phases. Each column represents the mean ± SEM of 7–8 animals/group, with a black dot showing individual mice. Asterisks indicate the levels of significance compared to the vehicle (control) group. ** *p* < 0.001 using one-way ANOVA.

In this set of experiments, we observed that treatment with *Spirulina* (30 mg/kg p.o.) inhibited the total time in different behavioral states during the inflammatory phase of the test, thus demonstrating a significant reduction in nocifensive behavior when compared to the control group, with an inhibitory rate of 60.64% (Figure 3). Moreover, treatment with indomethacin (10 mg/kg) decreased the time in different behavioral states during the inflammatory phase of the test in phase II compared to the control group, with a 57.06% inhibition of nocifensive behavior (Figure 3). This data collection demonstrates that *Spirulina* and indomethacin effectively prevented the inflammatory pain behavior induced by formalin when tested at the same treatment schedule.

### 3.4. Assessment of Locomotor Activity

**Figure 4:**
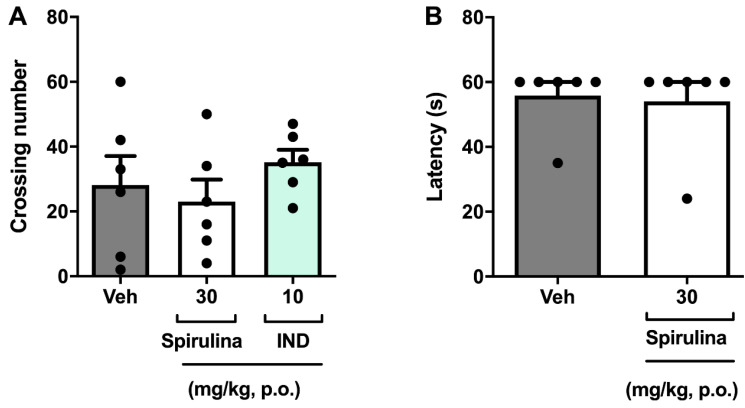
Effect of *Spirulina* in the open field test (panel **A**) and the rotarod test (panel **B**). Each column represents the mean ± SEM of 6 animals/group, with a black dot showing individual mice using one-way ANOVA. IND: indomethacin.

In another set of experiments, it was analyzed whether *Spirulina* could induce changes in mice’s locomotor performance during the antinociceptive effect to exclude probable false-positive results [46,47]. Herein, we evaluated the locomotor performance of mice treated with *Spirulina*. Relevantly, pre-treatment with *Spirulina* did not significantly affect the animals’ locomotor performance after oral administration of 30 mg/kg in the open field test (Figure 4A). Likewise, there was no deficit in locomotor function in the rotarod test after the previous administration of *Spirulina* (30 mg/kg p.o.) when compared to the control group (Figure 4B). Thus, our data demonstrated that single oral administration of *Spirulina* at a dose of 30 mg/kg to male mice demonstrated good tolerability and low toxicity when evaluated on motor behavioral parameters.

### 3.5. Nociceptive Behavior Induced by Menthol, Cinnamaldehyde, and Capsaicin

**Figure 5:**
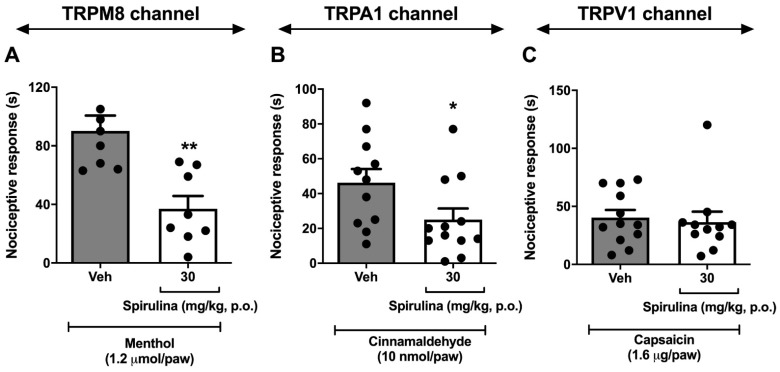
Effect of *Spirulina* on nocifensive behavior induced by menthol (panel **A**), cinnamaldehyde (panel **B**), and capsaicin (panel **C**). Each column represents the mean ± SEM of 7–12 animals/group, with a black dot showing individual mice. Asterisks denote the levels of significance compared to the control group (Veh). ** *p* < 0.001, * *p* < 0.05 using one-way ANOVA.

Then, the antinociceptive effects of *Spirulina* were evaluated in the tests of menthol (Figure 5A), cinnamaldehyde (Figure 5B), and capsaicin (Figure 5C). *Spirulina* (30 mg/kg, p.o.) significantly inhibited the nocifensive behavior induced by i.pl. menthol (TRPM8 channel) and cinnamaldehyde (TRPA1 channel), with 63.26% and 45.96% inhibitions respectively, compared to the control group. Capsaicin (TRPV1 channel) showed no significant difference when compared to the vehicle group. Taken together, it is possible to suggest that *Spirulina* has antinociceptive effects on ascending pain signaling for cold and/or inflammatory chemical stimuli through the TRMP8/TRPA1 pathway, although it did not show positive results in nociceptive pathways related to hot stimuli.

### 3.6. Nociceptive Behavior Induced by Glutamate

**Figure 6:**
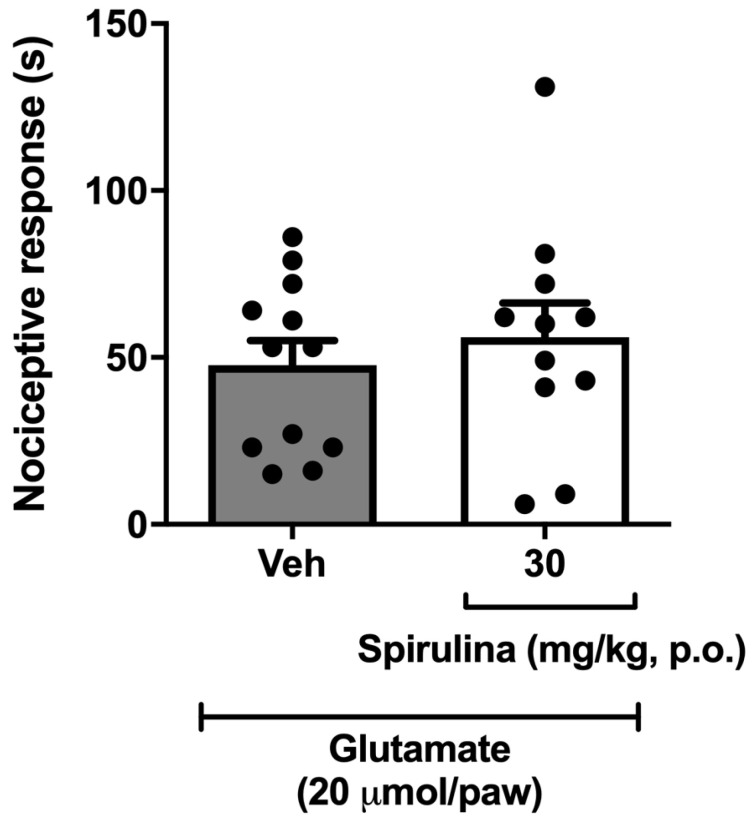
Effect of *Spirulina* on nociceptive behavior induced by glutamate in mice. Each column represents the mean ± SEM of 11–12 animals/group, with a black dot showing individual mice, using one-way ANOVA.

In the following protocol, it was observed that *Spirulina* (30 mg/kg, p.o.) did not show a significant reduction in the nocifensive behavior induced by i.pl. glutamate when compared to the vehicle group (Figure 6).

### 3.7. Assessment of Opioid System Involvement

**Figure 7:**
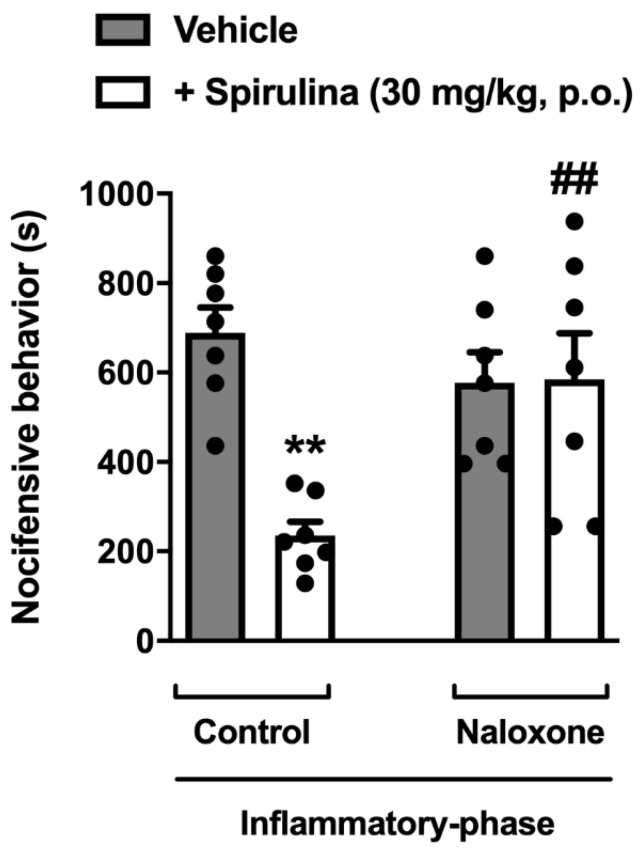
Involvement of opioid receptors in the antinociceptive effect of *Spirulina*. *Spirulina* (30 mg/kg, p.o.) or vehicle (0.9%NaCl 10 mL/kg) were administrated in mice previously treated with naloxone (1 mg/kg, i.p., a non-selective opioid receptor antagonist) during formalin-induced nocifensive behavior. Each column represents the mean ± SEM of 7 animals/group, with a black dot showing individual mice. Asterisks denote the levels of significance compared to the control group. ** *p* < 0.001, ## *p* < 0.001 using one-way ANOVA.

In this set of experiments, we investigated whether the treatment with *Spirulina* could inhibit the inflammatory pain through opioid-dependent mechanisms in mice. Relevantly, pre-treatment with a non-selective opioid receptor antagonist naloxone (1 mg/kg, i.p.) significantly reversed the antinociception induced by *Spirulina* in formalin-induced nocifensive behavior (Figure 7). These data, allied to those presented before, allow us to suggest that the analgesic effect showed by *Spirulina* upon inflammatory response might be related, at least in part, to its ability to modulate the TRPM8/TRPA1 channel and endogenous opioids signaling pathways expressed in the primary sensory neuron. However, additional protocols are needed to clarify whether *Spirulina* could affect descending pain control systems, such as monoaminergic pathways.

## 4. Discussion

This study aimed to investigate the mechanisms underlying the antinociceptive effects of *Spirulina*. Our data showed that *Spirulina* mitigated central sensitization induced by formalin injection and interacted with TRP channels to induce its antinociceptive effects, specifically, TRPM8 and TRPA1, which play a critical role in detecting environmental cold temperatures and mechanical sensations [48,49]. Our results also demonstrated the possible involvement of the opioid system as another mediator of *Spirulina*’s analgesic effect. As mentioned previously, *Spirulina* is a microalga with high nutritional and medicinal properties [10]. Herein, we used aceticacid-induced writhing response to demonstrate that a single oral treatment with *Spirulina* showed peripheral analgesic effects, in agreement with a previous study by Abu-Taweeland colleagues [28]. According to the authors, the antinociceptive effects of *Spirulina* (300 mg/kg) in rats submitted to the hot plate test were similar to a standard drug (morphine, 5 mg/kg) [28], suggesting that *Spirulina* seems to have great potential for its employment as a natural analgesic strategy.

Formalin test is a widely spread method of injury-produced inflammatory pain, quite often used to screen novel compounds and considered a more satisfactory clinical pain model. Through this test, our group demonstrated that *Spirulina* could be an exciting approach to managing clinical pain [50]. A biphasic response characterizes the formalin test, like phase I comprises the direct activation of primary nociceptive afferents by formalin (5–10 min), while phase II encompasses afferent activation produced by inflammatory mediators released following tissue injury pain, possibly leading to central sensitization (15–60 min). Herein, *Spirulina* only decreased the nociceptive behavior on phase II, similarly to other analgesic/anti-inflammatory drugs, such as gabapentin and indomethacin. Previously, Abdel-Daim and colleagues reported that STZ-diabetic rats orally treated with *Spirulina* in a 500 mg/kg/day dose for one month showed lower pain scores during both experimental phases [25]. Herein, it is necessary to highlight that the studies are very different concerning the methodology and dose used, which could justify the findings’ divergence. Altogether, it appears that the use of *Spirulina* could be a viable, natural, and low-cost alternative with high nutritional potential, which can become an adjuvant in the treatment of clinical pain. The activation of TRPA1 has been widely implicated as an essential pain transducer, and for this reason, the formalin test demonstrates important mechanisms for nociceptive understanding. Particularly during phase II, where the continuous and disseminated diffusion of formalin occurs along the nerves that express TRPA1 and lead to the release of a wide variety of different inflammatory mediators that can sensitize TRPA1 [51]. For this reason, the present study makes it possible to demonstrate that *Spirulina* through inhibition of TRPA1 could contribute to the control of neuropathic and inflammatory pain models [52].

Herein, during our experimental protocols, *Spirulina* induced its antinociceptive effects in an optimal level that does not follow a dose–response effect, possibly because it has reached its maximum effect. A similar effect is commonly observed with plant-based therapeutics, which show huge variability in the concentration of active compounds, resulting in therapeutic or side effects changes. Additionally, oral treatment with *Spirulina* (3, 10, 30, and 300 mg/kg) significantly inhibited the number of abdominal writhing induced by acetic acid. Regression analysis showed that *Spirulina* has an ED_50_ of 20.51 mg/kg and reached the maximal effect (E_max_) at a dose of 254.60 mg/kg, as evidenced in the dose–effect graphic (Figure 2). Based on these beneficial effects, a dose of 30 mg/kg of *Spirulina* was used in subsequent experiments to investigate some of the mechanisms underlying its antinociceptive effects. Abu-Taweeland collaborators demonstrated similar results in the test of abdominal contortions induced by acetic acid and in the control of thermal nociception with a dose of 200 mg/kg [28]. However, it is noteworthy that the authors chose to use a single dosage to compare the results, limiting the determination of a nociceptive response dose profile with the administration of *Spirulina*. Furthermore, Chamorro and colleagues evaluated the neuroprotective effects of *Spirulina* (25, 50, 100, 150, 200 mg/kg) in a Parkinson’s model. The authors reported that treatment for fourteen days with *Spirulina* (only at the dose of 150 mg/kg, p.o.) reduced dopamine depletion by 51% in mice that received 1-Methyl-4-phenyl-1,2,3,6-tetrahydropyridine hydrochloride (MPTP) administration [53].

Knowing that functional foods have a wide range of bioactive ingredients, which can modulate the activity of multiple cellular pathways, next, we decided to investigate the mechanisms of action through which *Spirulina* mediates its analgesic effects. The effect of *Spirulina* against menthol- and cinnamaldehyde-evoked pain is of great interest because both agonists play an important role during nociceptive processes. From our experiments, it was possible to identify that *Spirulina* interacts with TRP channels—particularly TRPM8 and TRPA1—to inhibit nociceptive behavior. In turn, TRPA1 is mainly located in nociceptive neurons of the peripheral nervous system (PNS) and, for this reason, is considered a target for anti-inflammatory and analgesic therapies [48,54]. To our knowledge, this is the first study to examine the interaction of *Spirulina* with TRP channels. Despite this, according to Horvath and colleagues, lutein—a natural dietary carotenoid—inhibited the activation of TRPA1 receptors on capsaicin-sensitive peptidergic sensory nerve endings and the consequent neurogenic and non-neurogenic inflammatory responses [55]. Interestingly, *Spirulina* is naturally rich in pigments, chlorophyll, phenolic compounds, such as carotenoids, and phycocyanin [10]. Additionally, in the present study, we investigated the phenol contents and scavenging potential of *Spirulina*, evaluated by investigating their DPPH reduction against the positive control. Our data demonstrated a significant relationship between phenolic content, particularly gallic acid, chlorogenic acid, quercetin, and catechin contents, and DPPH scavenging, as well as antioxidant activity. Relevantly, our data are also in accordance with the outcomes of different research groups that reported a positive correlation between total phenolic content, including flavonoids, and antioxidant activity [32,35,56,57,58]. Likewise, Bierhals et al. and Machado et al. reported a similar result between the phenolic content and the radical scavenging activity of *Spirulina* extracts [30,31]. Thus, we hypothesize that the antinociceptive effect demonstrated by *Spirulina* through TRPA1 could be associated with the presence of phenolic, particularly chlorogenic acid and quercetin, in its composition [10], although additional experiments will be critical to confirm this hypothesis. In agreement, phenolic compounds have been recognized by their antioxidant properties and dietary intake related to decreased cancer, cardiovascular, and eye disease risk [59,60]. Another interesting point to be highlighted is the presence of gallic acid among the constituents of *Spirulina*. Our data suggest that the radical scavenging effect is related to the type of phenolic compound (i.e., gallic acid and chlorogenic acid) and flavonoids (quercetin and catechin) present in the samples. In this sense, it was established that gallic acid has the potential to reduce the influx of calcium mediated by the activation of TRPA1, and for this reason, it shows an antinociceptive effect by acting as a TRPA1 antagonist [61]. For this reason, we believe that the antioxidant and analgesic effects related to TRPA1 inhibition found in the present study may be associated with the presence of gallic acid in *Spirulina*.

Similarly, TRPM8 is a non-selective cation channel activated by cold temperature and by cooling agents (for instance, menthol, eucalyptol, and icilin), recognized by its role in pain transduction since it is mainly expressed in a subpopulation of cold-sensitive dorsal root ganglion (DRG) neurons and sensory nerves [62,63]. Consistent with our findings, a prior study also reported that TRPM8 antagonists—DFL23693 and DFL23448—can decrease the nociceptive response during formalin-induced orofacial pain and in rats exposed to chronic constriction injury-induced neuropathic pain [64]. In accordance, the systemic administration of 1-phenylethyl-4-(benzyloxy)-3methoxybenzyl(2-aminoethyl)carbamate (PBMC)—a TRPM8 antagonist—significantly reduced the cold hypersensitivity in inflammatory and nerve-injury pain models [65]. Consequently, we can also hypothesize that TRPM8 channels possibly contribute to modulating the antinociceptive activity of *Spirulina*. Additionally, knowing the role of TRPM8 in pain hypersensitivity (characterized by cold allodynia and cold hyperalgesia) [63,65,66,67,68], it is possible to theorize about the beneficial effects of *Spirulina* for the management of pain hypersensitivity. Nevertheless, as previously mentioned, further studies are required in order to corroborate our findings.

Regarding our data of capsaicin- and glutamate-induced elevating, licking, biting, or shaking the injected paw or reducing the weight put on it, *Spirulina* did not show significant antinociceptive activity. Glutamate is the primary excitatory neurotransmitter in adult mammals’ nervous systems and plays a significant role in nociceptive processes (acute and chronic pain) [69,70]. Its intra-plantar injection results in mechanical and thermal hyperalgesia because this neurotransmitter can excite and sensitize peripheral axons of primary afferent neurons, binding to ionotropic and metabotropic receptors [71]. At the same time, capsaicin (a chili pepper extract) produces painful sensations upon cutaneous application by activating the transient receptor potential cation channel subfamily V member 1 (TRPV1) [72,73]. It is recognized as an ion channel predominantly expressed by a subset of peripheral sensory neurons involved in pain sensation [74]. Both tests are among the main nociception models validated and extensively performed in rats and mice [75]. Through these tests, we can infer that glutamate receptors and TRPV1 channels possibly are not involved and do not contribute to the modulation of antinociceptive effects of *Spirulina*. However, more accurate data from bioinformatics or pharmacological approaches will be needed to confirm thesepropositions.

Since the opioid receptors and their ligands produce potent analgesia, the endogenous opioid system has a central role in pain [76,77]. Naloxone, in turn, is a non-selective, short-acting opioid receptor antagonist commonly used to treat these overdoses in clinical practice [78]. Herein, we demonstrated that naloxone reversed the antinociceptive effect of *Spirulina* during the formalin test. Following our findings, Santos and colleagues reported that the treatment of mice with orally supplied *Spirulina* LEB-18 dried biomass (*Spirulina*-LEB18) (50–400 mg/kg) 1 h before intra-plantar injection of Freund’s Complete Adjuvant (CFA) prevented the CFA-induced allodynia in a dose-dependent manner [79]. As in our work, the antinociceptive effect induced by *Spirulina*-LEB18 (200 mg/kg) was reduced by naloxone administration during a tail-flick test. It should also be noted that the efficacy of *Spirulina*-LEB18 (200 and 400 mg/kg) in the tail-flick test was quite similar and lasted longer than that induced by morphine [79]. Another critical component present in *Spirulina* was quercetin, and it may also be associated with analgesic effects from the activation of the opioid system found in the present study. Relevantly, previous reports demonstrated that quercetin reduced the inflammatory response through activation of the opioid system, with consequent inhibition of (i) neuronal adenylate cyclase, (ii) prostaglandins levels, and (iii) hyperalgesia [80,81]. Taken together, our data suggest that the antinociceptive effect of *Spirulina* could be attributed to the single or synergic action of these main phenolic components or even other minor constituents present in the blue-green algae. Nonetheless, additional studies are necessary to test this hypothesis. These findings suggest, at least in part, that the analgesic effect of *Spirulina* seems to be mediated by the opioid system. Relevantly, we did not find evidence that *Spirulina* affects motor performance evaluated by spontaneous locomotion in the open field test and rotarod. It is a commonly used test to assess mice’s locomotor and behavioral activity levels, allowing for evaluating drug-related effects on different aspects of animal behavior [82,83]. In agreement with our results, Santos and colleagues also reported that *Spirulina*-LEB18 (400 mg/kg) did not affect mice’s motor function exposed to the rotarod performance test [63]. In this way, *Spirulina* seems to mediate its antinociceptive effects without inducing motor impairment or sedation.

The limitation of our study was the lack of control for algogenic injection (i.e., absolute control with the injection of the vehicles of each algogenic), which would be essential to evaluate how the behavior of animal’s changes compared to the absolute control and consequently, observe some partial effects. However, we and others have previously published reports related to algogenic injection with the same methodology used in this study [44,84,85,86,87,88,89,90]. Moreover, the number of animals and the intensity of noxious stimuli used were the minima necessary to demonstrate consistent effects. All of the experimental procedures were conducted according to the guidelines of CONCEA and CEUA/UFSC, based on the principles of the 3Rs (Replacement, Reduction, and Refinement). Finally, due to the current coronavirus COVID-19 pandemic—a global health crisis—that affected the routine of the university, further investigations are permanently suspended, making it unfeasible to conduct additional experiments.

## 5. Conclusions

**Figure 8:**
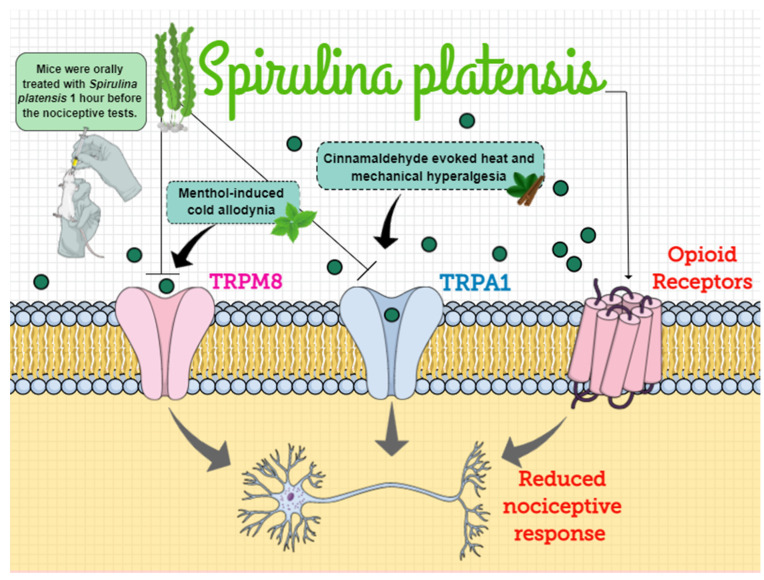
The mechanisms of action behind the antinociceptive effects of *Spirulina platensis*. *Spirulina* belongs to a group of aquatic organisms named blue-green algae and to the phylum Cyanobacteria. Considering that the use of this algae is widespread among people due to its diverse biological activities, herein, we demonstrated the possible mechanism of action by which *Spirulina* could exert its antinociceptive effects in mice submitted to different models of acute nociceptive response. Our results demonstrated that *Spirulina* markedly decreased cold-allodynia, heat, and mechanical hyperalgesia through inhibition of TRPM8 and TRPA1 ion channels’ activation by their respective agonists—menthol and cinnamaldehyde. Additionally, we also reported that naloxone reversed the antinociceptive effect of *Spirulina* during the formalin test, which suggests that the analgesic effect of *Spirulina* seems to be mediated, at least partially, by the opioid system. Thus, it is possible to hypothesize that *Spirulina* exerts antinociceptive effects during inflammatory models by modulation of TRPM8/TRPA1 channels and endogenous opioids signaling pathways. TRPM8: transient receptor potential cation channel subfamily M (melastatin) member 8; TRPA1: transient receptor potential ankyrin 1 channel.

*Spirulina* has been recognized in the literature as a functional food due to its high nutritive value, and previous studies have demonstrated its beneficial health effects linked to potent free-radical scavenger [91], anti-inflammatory [28], and analgesic properties [25]. The present study highlighted the antinociceptive effect of a single treatment with *Spirulina* in mice submitted to different models of acute nociceptive response and its underlying mechanisms of action related to the ascending pain control and antioxidant effect. Altogether, we demonstrated that *Spirulina* exerts antinociceptive effects during inflammatory models by modulating TRPM8/TRPA1 channels and endogenous opioids signaling pathways. In this way, considering that dietary supplements’ use is considered safe and approved by the Food and Drug Administration (FDA), *Spirulina* could represent a natural analgesic strategy and adjuvant for treatment of different inflammatory clinical pain (Figure 8).

## Figures and Tables

**Table 1:** Constituents of *Spirulina platensis*. Average nutritional analysis per 100 g.

Nutritional Values	Per 100 g	* RIA/** NRV
Energy	1364 kJ/326 Kcal	
Lipids	1 g	
-of which saturated	0.5 g	
Carbohydrates	13.1 g	
-of which sugars	<0.1 g	
Fiber	5.1 g	
Proteins	65.9 g	133% *
Salt	0.9 g	
Potassium	1040 mg	52% **
Calcium	332.5 mg	42% **
Iron	83.2 mg	594% **
Vitamin B6 (Pyridoxine)	18.5 mg	1314% **
Vitamin B12 (Cobalamin)	170 μg	6800% **
Vitamin E	12.7 mg	105% **

* Reference intake for an average adult (8400 kJ/2000 Kcal); ** Nutrient reference value.

**Table 2:** Total phenolic compounds, flavonoids content, and antioxidant activity of *Spirulina platensis*.

*Spirulina platensis*				
Phenolic compounds (μg/mL)				Mean ± SD
-Gallic acid	100.89	102.95	102.05	102.0 ± 1.03
-Chlorogenic acid	125.15	141.25	125.65	130.7 ± 9.15
Flavonoids contents (μg/mL)				
-Quercetin	624.23	611.42	605.00	613.6 ± 9.79
-Catechin	230.19	230.77	207.31	222.8 ± 13.38
Antioxidant activity				
-Trolox (μg/mL)	132.76	120.86	140.86	131.5 ± 10.06
-Scavenging activity (%)	42.17	40.16	43.55	41.96 ± 1.70

## Data Availability

All the results used in this work to support the conclusions of this study are included in the article.
